# Lung Microtissue Array to Screen the Fibrogenic Potential of Carbon Nanotubes

**DOI:** 10.1038/srep31304

**Published:** 2016-08-11

**Authors:** Zhaowei Chen, Qixin Wang, Mohammadnabi Asmani, Yan Li, Chang Liu, Changning Li, Julian M. Lippmann, Yun Wu, Ruogang Zhao

**Affiliations:** 1 State University of New York at Buffalo, Department of Biomedical Engineering, Buffalo, New York, 14260, USA; 2 State University of New York at Buffalo, Department of Chemical and Biological Engineering, Buffalo, New York, 14260, USA

## Abstract

Due to their excellent physical and chemical characteristics, multi-wall carbon nanotubes (MWCNT) have the potential to be used in structural composites, conductive materials, sensors, drug delivery and medical imaging. However, because of their small-size and light-weight, the applications of MWCNT also raise health concerns. *In vivo* animal studies have shown that MWCNT cause biomechanical and genetic alterations in the lung tissue which lead to lung fibrosis. To screen the fibrogenic risk factor of specific types of MWCNT, we developed a human lung microtissue array device that allows real-time and *in-situ* readout of the biomechanical properties of the engineered lung microtissue upon MWCNT insult. We showed that the higher the MWCNT concentration, the more severe cytotoxicity was observed. More importantly, short type MWCNT at low concentration of 50 ng/ml stimulated microtissue formation and contraction force generation, and caused substantial increase in the fibrogenic marker miR-21 expression, indicating the high fibrogenic potential of this specific carbon nanotube type and concentration. The presented microtissue array system provides a powerful tool for high-throughput examination of the therapeutic and toxicological effects of target compounds in realistic tissue environment.

Due to their excellent physical and chemical characteristics, multi-wall carbon nanotubes (MWCNT) have the potential to be used in structural composites, conductive materials, sensors, drug delivery and medical imaging^1^,^2^,^3^,^4^. However, because of their small-size and light-weight, MWCNT expose to the working environment as particular matter (PM) of respirable size, and are considered as an occupational inhalation exposure risk^5^. The fiber-shape of MWCNT which is similar to asbestos reminded people the potential of MWCNT for inducing pulmonary diseases, especially idiopathic pulmonary fibrosis (IPF)^6^,^7^,^8^. *In vivo* animal studies have shown that MWCNT with different length^9^, diameter^10^, contaminants^11^ and surface modification^12^ cause cellular apoptosis and inflammation in the lung, which lead to the thickening and stiffening of the lung tissue^5^,^6^,^13^,^14^,^15^,^16^,^17^,^18^. Such elevation of the mechanical properties of the tissue is a known hallmark of tissue fibrosis. Therefore, identifying the impacts of specific types of MWCNT on the physiological conditions of lung tissue including mechanical properties is important to understand the health safety of carbon nanotubes. However, due to the high cost and low throughput of the animal models, the progress in this field is very slow. An experimental system that can allow easy monitoring of the physiological conditions of the lung tissue after MWCNT exposure is greatly needed.

Recently emerged bio-microelectromechanical systems (bio-MEMS) have been shown as a viable solution to provide low-cost and high-throughput readout of the biochemical and biomechanical conditions of the cells and tissues. Micropost arrays made of polydimethylsiloxane (PDMS) have been used to measure the cellular contraction force and to regulate stem cell differentiation^19^,^20^. Microfluidic channel systems were also developed to study the impact of fluidic shear flow on the physiology of endothelium^21^,^22^ and cancer cell invasion into surrounding tissue^23^. We have previously developed a microtissue force gauge to measure the collective contraction force of several hundred cells as they self-assemble into a microtissue of less than 1mm in size^24^. Due to the small size of each microtissue and the array format of the device, this system is well suited for low cost and rapid study of the physiological conditions of engineered tissues.

Based on the microtissue force gauge, we report in this study the development of an engineered human lung microtissue array system for the real-time, *in situ* monitoring of the biomechanical impacts of MWCNT on lung cells and tissues. The inherent morphogenic nature of the system allowed us to model the structure of human lung epithelial tissue. Taking advantage of the large array format and the *in-situ* force measurement capacity of our microtissue device, we performed rapid, real-time measurement of the changes in the mechanical properties of BEAS-2B (B2B) normal lung epithelial cell-populated microtissues. B2B cells were challenged with two types of MWCNT. One is pristine long type MWCNT 10–50 μm in length with no surface modification, and the other is short type MWCNT 0.5–2 μm in length with carboxylate-modified surface (S-MWCNT-C). We showed that high concentration carbon nanotube treatment caused severe cytotoxicity. Short type carbon nanotube at low concentration of 50 ng/ml stimulated microtissue formation and contraction force generation and caused substantial increase in the fibrogenic marker miR-21 expression, indicating the high fibrogenic potential of this specific carbon nanotube type and concentration. These data demonstrated the screening capability of the microtissue array system. This microtissue array device represents a powerful tool to allow rapid examination of the pharmacological impact of target compounds in physiologically-relevant tissue environment. Insights gained from this study may assist in understanding the development of pulmonary diseases induced by MWCNT.

## Results

**Figure 1:**
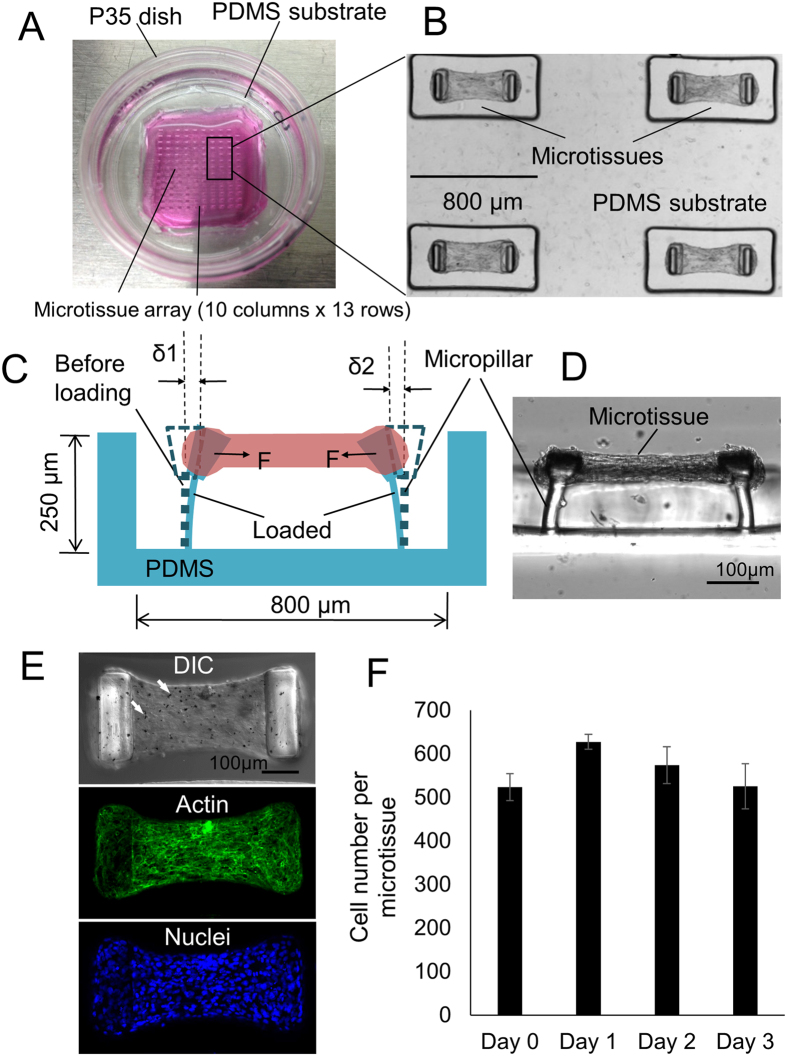
Overview of the engineered lung microtissue array device. (**A**) A P35 petri-dish contains a microtissue array fabricated in a PDMS substrate. (**B**) A portion of the microtissue array (2 × 2). (**C**) Schematic sideview of a microtissue hanging between two micropillars. Microtissue contraction force was determined by the deflection of the micropillars. (**D**) Actual sideview of a microtissue. (**E**) DIC image and Z-projected confocal images of actin and nuclei of the microtissue. The cells were treated with 5 μg/mL S-MWCNT-C before encapsulated in this microtissue. Agglomerates of carbon nanotube trapped inside the microtissue were visible (Arrows). (**F**) Cell proliferation inside the microtissue over a 3 days period.

We fabricated the microtissue array device by PDMS replica molding from masters made using a multilayer microlithography technique as previously described^25^. Each device consists of arrays of PDMS microwells (10 × 13) with each microwell containing a self-assembled microtissue hanging between a pair of micropillars (Fig. 1A,B,D). The micropillar pair in each microwell not only provides mechanical support to the microtissues but also serves as an *in-situ* force gauge to measure tissue contraction forces. Since it is well established that fibrotic tissues *in vivo*, such as those at the edges of a closing wound, generate elevated contraction force, the contraction force can be used as a phenotypical indicator for fibrotic tissue. Utilizing this property, we screened the fibrogenic potential of the MWCNT by comparing the differences in microtissue force generation upon various MWCNT treatments. We treated B2B normal lung bronchial epithelial cells before microtissue seeding with MWCNT and S-MWCNT-C at concentration of 50 ng/mL, which was calculated based on the dosages used in animal studies to mimic *in vivo* situation^26^,^27^,^28^. In addition, we challenged B2B cells with S-MWCNT-C at much higher concentration, 5 μg/mL, which was based on IC50 measurement on cell viability *in vitro* and represents the acute exposure of carbon nanotubes. Treated B2B cells and unpolymerized type-I collagen were introduced to the microwells through centrifugation. No carbon nanotubes were added after cell seeding. Several hours after seeding, the collective contraction of the cells started to compact the collagen matrix in individual microwells. We observed the formation of dog-bone shaped, aligned microtissues hanging between the heads of a pair of micropillars 12–24 hours after seeding (Fig. 1D). Large agglomerates of carbon nanotubes inside the microtissue were only visible for the highest concentration treatment (5 μg/mL) of S-MWCNT-C under light microscopy (Fig. 1E). Cells embedded in the collagen matrix of the microtissue showed an elongated morphology with F-actin stress fibers running along the longitudinal axis of the microtissues (Fig. 1E). Such well-organized cytoskeletal morphology is consistent with the generation of axial contractile forces by the cell population observed at the whole microtissue level. Embedded cells also expressed tight junctions (ZO-1/TJP1) that are known to predominantly express in epithelial cells (Fig. S1). Confocal microscopy analysis showed that embedded cells formed nearly a monolayer inside the microtissue (Fig. S2). The deflection of the micropillars was detected using optical microscopy and was used to calculate the spontaneous microtissue contraction force according to cantilever bending theory (Fig. 1C,D). Microtissues were stable over a period of one week without detaching from the micropillar heads. Cell proliferation in the microtissue was not significant (Fig. 1F), consistent with previous findings for embedded cells in collagen matrix^29^,^30^.

**Figure 2:**
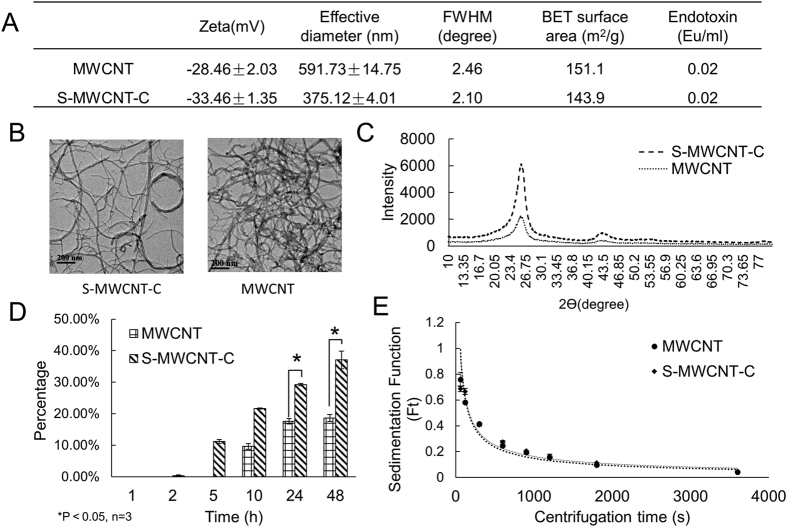
MWCNT characterization. (**A**) Zeta potential, diameter, FWHM, BET, and endotoxin measurement of MWCNT. (**B**) TEM images of MWCNT. (**C**) XRD measurement of MWCNT shows two types of MWCNT have similar structure and there was little contaminant. (**D**) B2B cellular uptake of MWCNT. S-MWCNT-C showed higher uptake percentage than long type MWCNT. (**E**) Sedimentation measurement for MWCNT.

We analyzed the physical characters of the carbon nanotubes using various methods including zeta potential measurement, dynamic light scattering (DLS), x-ray diffraction (XRD), BET surface analysis (BET) and the transmission electron microscopy (TEM). As shown in Fig. 2A, MWCNT showed less negative surface charge and larger effective diameter than S-MWCNT-C. Transmission electron microscope images showed tubular shape for both types of carbon nanotubes and the lengths were around 0.5–2 μm and 10–50 μm for S-MWCNT-C and MWCNT, respectively (Fig. 2B). In XRD measurement, the full width at half maximum (FWHM) of MWCNT was similar to that of S-MWCNT-C, indicating similar crystallinity. XRD spectrum also showed similar structure between two types of carbon nanotubes with little contaminants (Fig. 2C). In XRD profiles, a sharp and strong diffraction peak at around 2θ = 25.8 degree and a broad and much shorter peak centered at 2θ = 43.0 degree was observed, corresponding to the (002) and (100) Bragg reflection of hexagonal graphite structure. These X-ray diffraction patterns indicated that all carbon nanotube samples possess a main feature similarity to that of graphite crystal (Fig. 2C). For cellular uptake, B2B cells started to uptake S-MWCNT-C at 5h and MWCNT at 10 h post exposure. At 24 h post exposure S-MWCNT-C was uptaken by about 30%, significantly higher than the 17.5% for MWCNT. At 48 h post exposure, S-MWCNT-C was uptaken by 37% while there was no change in the uptake for MWCNT (Fig. 2D). The sedimentation test for both type carbon nanotubes showed similar sedimentation rates (Fig. 2E). We also performed endotoxin test on all materials used in this study, including two types of carbon tubes, cell culture medium, collagen and buffer solutions. Both carbon nanotubes showed very low endotoxin level (0.02 Eu/ml), which is much lower than the FDA limit of 0.25 Eu/ml, all other materials were below the detection limit.

**Figure 3:**
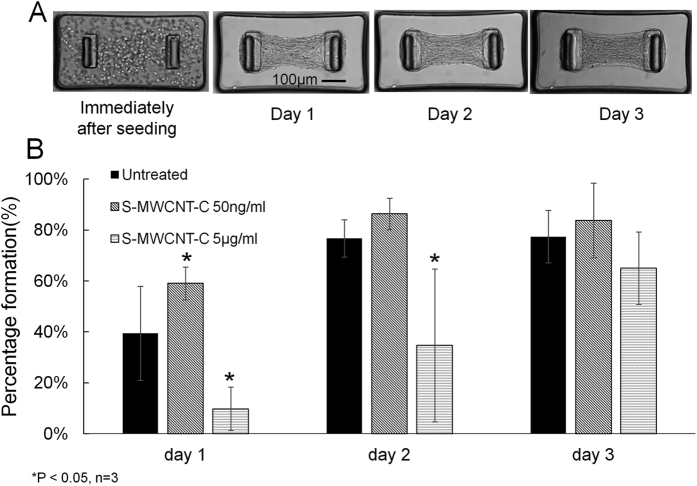
The impact of carbon nanotube treatments on microtissue formation process. (**A**) The spontaneous contraction force of the embedded cells caused the compaction of the collagen gel and the formation of microtissues over a 3 days period. (**B**) Low concentration S-MWCNT-C treatment stimulated microtissue formation whereas high concentration of the same type carbon nanotube substantially inhibited microtissue formation over a 3 days period as compared to untreated control.

We then investigated the effects of MWCNT exposure on the microtissue formation process. As shown in Fig. 3, the treatment of S-MWCNT-C at low concentration of 50 ng/mL caused substantially increased number of microtissue formation in the device through a 3 days period as compared to the control (untreated). However, the treatment of S-MWCNT-C at high concentration of 5 μg/mL substantially delayed the formation of microtissues during the same period of time. Since the microtissue formation is driven by the spontaneous contraction of the embedded cell population, the above data suggested that low concentration S-MWCNT-C treatment at 50 ng/mL enhanced cell contraction force but high concentration S-MWCNT-C treatment at 5 μg/mL inhibited cell contraction force.

**Figure 4 f4:**
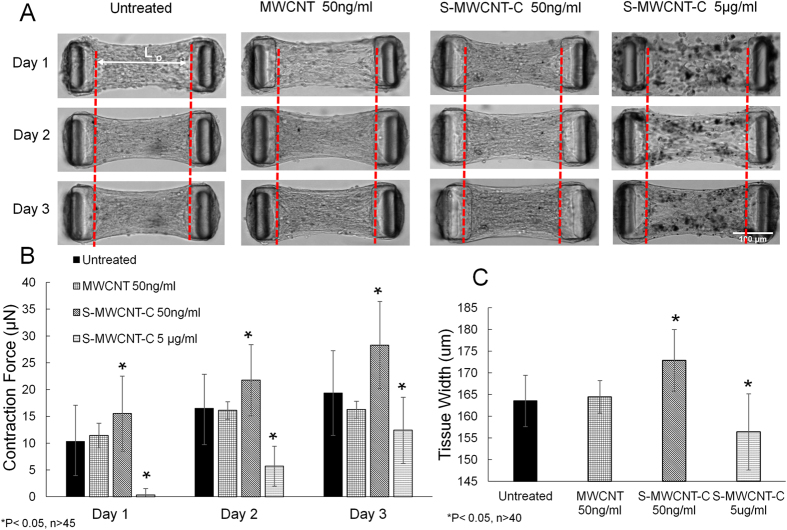
The impact of different carbon nanotube treatments on microtissue contraction force generation. (**A**) The evolving morphology of microtissues under different treatments during a 3 days period. Red dashed lines showed the position of the inner edge of the micropillar head on day 1. Micropillar heads moved towards the center as a result of increased contraction force at days 2 and 3. (**B**) Contraction force measurement. S-MWCNT-C at 50 ng/ml caused substantial increases in contraction force generation. (**C**) Microtissue width measurement at day 3.

To further understand the impact of MWCNT treatment on microtissue force generation, we monitored the contraction forces of well-formed microtissues under various treatments over a 3 days period. The contraction forces continued to increase for all groups over a 3-day culture period with the S-MWCNT-C 50 ng/ml group producing the highest contraction force. As shown in Fig. 4A[Fig f4]B, the contraction force increased from 15.5 μN to 21.7 μN between day 1 and day 2 in S-MWCNT-C 50 ng/ml treated group, and continued to increase another 6.5 μN by day 3. In addition, the microtissues in this group were shorter along the longitudinal axis but larger in the lateral width. This is due to the high contraction force along the longitudinal direction that caused the compression of the microtissue in this direction and corresponding expansion in the lateral direction (Fig. 4A,C). The averaged microtissue width increased from 163.6 μm for untreated control group to 172.9 μm for S-MWCNT-C 50 ng/ml treated group (Fig. 4C). The MWCNT 50 ng/ml treated group had almost the same contraction force as the untreated control group, indicating little influence of MWCNT to the biomechnical properties of the microtissues. This is probably due to very limited uptake of this type of carbon nanotube by the cells as a result of their large dimension (Fig. 2D). Indeed, the length of the MWCNT used in the current study is about 10–50 μm, which is comparable to a single cell dimension and is approximately 25 times longer than S-MWCNT-C. For S-MWCNT-C 5 μg/ml treated group, although the contraction force and tissue width were significant lower than other groups due to possible cytotoxicity induced by the much higher dosage, the increase rate of contraction force was 12.8 folds higher than S-MWCNT-C 50 ng/ml group between day 1 and day 2, and 1.7 folds higher than S-MWCNT-C 50 ng/ml group between day 2 and day 3.

**Figure 5:**
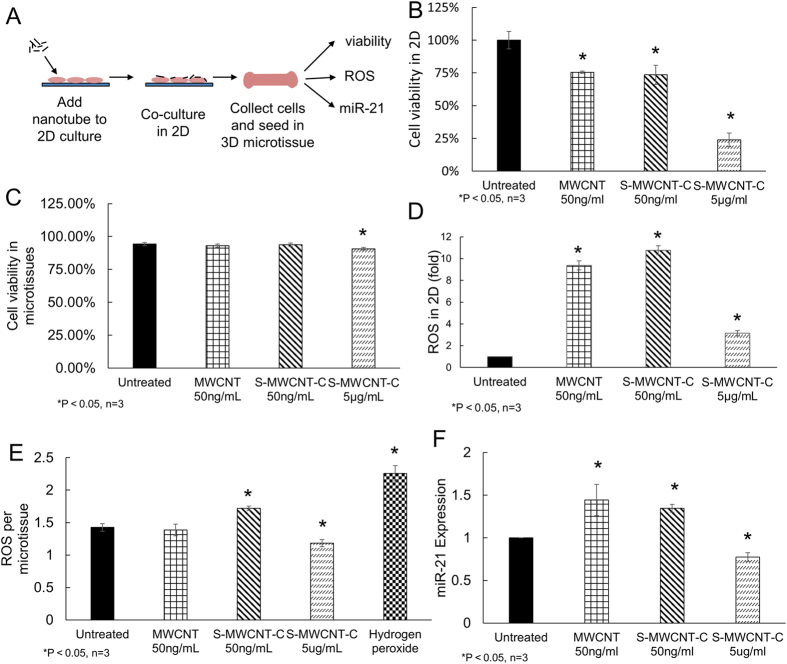
The impact of carbon nanotube treatments on cell viability, reactive oxygen species (ROS) and miR-21 expression. (**A**) Schematic procedure of carbon nanotube treatments for microtissue experiments. (**B**) Cell viability test in 2D culture after treatment with different carbon nanotube groups. S-MWCNT-C at 5 μg/ml caused severe cell death. The treatment of MWCNT and S-MWCNT-C at 50 ng/mL showed mild cytotoxicity. (**C**) Cell viability test in microtissues after treatment with carbon nanotubes. (D) ROS measurement in 2D culture; (**E**) ROS measurement in microtissue; (**F**) miR-21 expression after carbon nanotube exposure. The treatment of MWCNT and S-MWCNT-C at 50 ng/mL caused substantial increase in miR-21 expression level.

To understand the mechanism of contraction force regulation by MWCNT, we studied cytotoxicity of MWCNT both in 2D culture and in microtissues. In 2D culture, B2B cells were treated with MWCNT and S-MWCNT-C at low concentration of 50 ng/mL and with S-MWCNT-C at high concentration of 5 μg/mL respectively. At 48 h post exposure, significant decrease in cell viability was observed in all carbon nanotube treated groups as compared to untreated control, especially in the group treated with S-MWCNT-C at high concentration (Fig. 5B). The treatment with S-MWCNT-C at high concentration of 5 μg/mL caused more cell death than both types of MWCNT at low concentration (50 ng/mL) (Fig. 5B and Figure S3), which agreed with previous reports^31^,^32^. Meanwhile, significant increases in reactive oxygen species (ROS) were observed in both types of MWCNT treated samples (Fig. 5D), indicating ROS might be responsible for the substantial cell death. However, we did not observe higher ROS level in high concentration S-MWCNT-C treated condition, which is likely due to the substantial loss of cells caused by the acute toxicity.

We also evaluated the cytotoxicity in microtissues. We first exposed cells to carbon nanotubes for 24 hours in 2D culture, and then seeded the B2B cells in microtissues (Fig. 5A). Cell viability in the microtissues was measured 24 hours after microtissue seeding. We found high cell viability for all carbon nanotube treated conditions (>90%) in the microtissues, likely because only cells surviving 2D nanotube treatments remained attached and were trypsinzed and introduced into the microtissues (Fig. 5C and Figure S4). The ROS levels for MWCNT at low concentration (50 ng/mL) and S-MWCNT-C at high concentration (5 μg/mL) are comparable to that of the untreated control (Fig. 5E). The relatively low oxidative stress level in these microtissues would permit the survival of the cells. However, the ROS level for S-MWCNT-C at 50 ng/mL is significantly higher than that of the untreated control (Fig. 5E). Interestingly, such high level oxidative stress did not cause significant cell death. Instead it correlated well with the high contractile force generation in this treatment condition (Fig. 4B), which suggested that oxidative stress may trigger downstream pathways that are responsible for contractile force generation. In the future, we will further investigate this phenomenon.

We also used qRT-PCR to measure fibrosis-related biomarkers in the microtissues after MWCNT treatments. MicroRNAs (miR), a family of short endogenous noncoding RNAs, harbor critical functions in the initiation and progression of cancer and many other diseases^33^,^34^. MicroRNAs have been found to be involved in the development of pulmonary diseases such as lung fibrosis and lung cancer^34^,^35^. In this study, we measured the expressions of miR-21 at day 3 after carbon nanotube treatment in order to identify potential pathogenic pathways of lung fibrosis. To perform the qRT-PCR measurement, microtissues were first collected manually from the wells through repeated washing steps, and RNA was then extracted from the microtissues for miR-21 expression quantification. Results of qRT-PCR showed significant up-regulation of miR-21 in S-MWCNT-C 50 ng/ml groups (Fig. 5F). This is consistent with the high contraction force generation observed in the S-MWCNT-C 50 ng/ml group, which is another pathophysiological character of the fibrotic tissues (Fig. 4A,B)^36^. When B2B cells were exposed to S-MWCNT-C at high concentration (5 μg/ml), acute cytotoxicity was shown by the loss of large number of cells and the slow formation of microtissues. As expected, we observed significant down-regulation of miR-21 in this group.

## Discussion

The health risk of carbon nanotubes is a topic under intensive research lately^5^,^37^, but very few previous studies have approached the topic from the cellular and tissue biomechanics aspect. Since biomechanics has been shown as a key regulator of many physiopathologoical processes, such as the differentiation of stem cells^38^,^39^,^40^, metastasis of the cancer cells^41^,^42^ and fibrotic differentiation of epithelial cells^43^,^44^, studying the biomechanical impact of carbon nanotubes at both cellular and tissue levels will help to elucidate their potential health risks and assist in better understanding the disease mechanism. In this study, we developed a novel engineered lung microtissue array device to study the biomechanical impacts of carbon nanotubes on lung epithelial cells and lung tissues in a relatively rapid manner. Our data showed that low concentration of S-MWCNT-C caused strong contraction force generation in B2B cell populated microtissues, which was accompanied by elevated level of ROS and fibrogenic marker miR-21 expression. However, the same type of carbon nanotube at high concentration did not cause high contraction forces, likely due to the cytotoxicity effect caused by the overdose of S-MWCNT-C. In fact, our study using a series of S-MWCNT-C concentrations showed that increased concentration caused increased level of cell death (Supplemental Fig. S3). Low concentration of long type MWCNT did not cause significant changes in the microtissue contraction force, likely due to their limited cellular uptake as a result of the large dimension (Fig. 2D). In the current study, our discussion about the fibrogenic potential is principally restricted to carbon nanotubes. However, it should be noted that other nanomaterials such as silica quartz^45^,^46^ and asbestosis fibers^47^,^48^ have been reported to cause fibrogenic responses *in vivo*. It would be interesting to explore their responses in the microtissue array device in the future.

In the current study, we used B2B normal human bronchial epithelial cells to construct engineered lung microtissues. B2B cells are widely used as an *in vitro* model to investigate the relationship between engineered nanomaterials and lung diseases^49^,^50^,^51^ mainly due to their easiness to handle. Even though alveolar epithelial cells are the most relevant model for lung fibrosis due to the fact that majority of the fibrosis occurs in lung parenchyma, there is currently no commercially available healthy human alveolar epithelial cell lines. Cancerous alveolar epithelial cell lines, such as A549 cells, are not good choice for the current study due to their diseased state. In fact, we tested the microtissue formation using A549 cells and we found loose and non-stable tissue formed by A549 cells (Supplemental Figure S5), showing a significant difference compared to the healthy B2B cell line. In future studies, it would be ideal to include other supporting cell types such as macrophages and pericytes in the microtissue model since these cells have been found to contribute to the initiation and progression of lung fibrosis.

The elevation of the tissue contraction force by S-MWCNT-C treatment at 50 ng/mL is interesting and can be caused by several factors. Previous studies have shown that cellular exposure to the carbon nanotubes can influence the activity of the microtubules, such as damaging the mitotic spindle during cell division^52^,^53^. It has been proposed that since the tubular shape of the carbon nanotubes is inherently similar to that of the microtubule and the diameter (8–15 nm) of the carbon nanotubes is close to that of the microtubule, it is possible that the S-MWCNT-C uptaken by the cells replaced part of the tubulin units and interfered with the microtubule polymerization during dynamic organization of the cytoskeletal network^54^. Microtubules are a well-known cytoskeletal component that affects cellular mechanics, for example, preventing microtubule polymerization with Nocodazole caused the increase in cellular contraction force^55^,^56^ and disrupting microtubules into fragmented pieces induced larger cell contraction force^57^,^58^. In our study, S-MWCNT-C treatment at 50 ng/mL caused significant morphological change in B2B cells cultured in 2D. Cells formed long filopodia-like microtubule protrusions similar to the morphology of migrating cells (Supplemental Fig. S6). Therefore, the interference with the microtubule dynamics by S-MWCNT-C is a possible reason for the changes of tissue contraction forces.

Recently extensive research has been done to decipher the roles of microRNAs in the initiation and progression of many lung diseases. Increased miR-21 expression was observed in the lungs of patients with idiopathic pulmonary fibrosis. Up-regulation of miR-21 has been shown to be associated with the epithelial-mesenchymal transition (EMT) both *in vitro* and *in vivo* through the TGF-β/Smad signaling pathway^34^,^35^,^59^,^60^. In this study we showed that S-MWCNT-C treatment caused synchronized up-regulation of miR-21 and microtissue contraction force, which suggests that carbon nanotubes may induce the EMT and early fibrotic differentiation of the lung microtissues through microRNA regulation. However, we did not observe significant difference in TGF-β mRNA expression level between different treatment groups (Supplemental Fig. S7), indicating miR-21 may take effect through other signaling pathways than TGF-β/Smad, such as pathways related to oxidative stress because we did observe good correlation between the miR-21 expression and ROS levels.

In summary, we have developed a novel engineered microtissue array device that allows real-time and *in situ* characterization of the biomechanical impact of carbon nanotubes in lung microtissues. We found that high concentration carbon nanotube treatment caused severe cytotoxicity in 2D culture and delayed microtissue formation, but short type S-MWCNT-C at low concentration of 50 ng/ml stimulated microtissue formation and contraction force generation, and caused substantial increase in the ROS level and fibrogenic marker miR-21 expression, indicating the high fibrogenic potential of this specific carbon nanotube type and concentration. These data demonstrated the screening capability of the microtissue array system. This microtissue array device represents a powerful tool to allow rapid examination of the pharmacological impact of target compounds in engineered tissue environment and can be used to model several mechanosensitive tissue types including skeletal and cardiac muscles, skins and tendons and ligaments. It is expected that the utilization of this system will lead to more robust and higher throughput screening of the disease mechanisms and treatments. The current system has limited capacity in handling multiple biochemical conditions simultaneously. Future improvement such as integrating the device with multiwall plates will enhance the biochemical experimental throughput of the system.

## Methods

### Microtissue Array Device Fabrication

We used a multilayer microlithography technique to fabricate the SU-8 master of the microtissue array device, as previously described^25^. Briefly, a first layer of SU-8 for the leg section of the micropillar was placed on the silicon wafer and exposed. The layer for the head section was then placed on top of the leg layer and the enlarged head pattern was aligned with the leg pattern and exposed. A blocking photoresist layer in between the leg and head layers was used to prevent over-exposure of the leg section during head section exposure. Micropillar array pattern was then transfered to polydimethylsiloxane (PDMS, Dow-Corning, Sylgard 184) stamp via replica molding. The final device was casted in a P35 petri-dish using PDMS stamps for the ease of handling (Fig. 1A,B).

### MWCNT Preparation and Treatment

MWCNT and S-MWCNT-C were purchased from Cheap Tubes Inc. The diameters of both tubes were 8–15 nm. The length of MWCNT was 10–50 μm, and the length of S-MWCNT-C was 0.5–2 μm. Both carbon nanotubes were at least 95% purity. The carbon nanotubes were suspended in the dispersion medium (DM) which contained 0.01 mg/mL of 1,2-dipalmitoyl-sn-glycero-3-phosphocholine (DPPC), 0.6 mg/mL bovine serum albumin (BSA) and 5.5 mM D-glucose^61^. Before treatment, both carbon nanotubes were sonicated for 5–10 mins until there were no visible aggregates. B2B cells were seeded into 6-well plates at the density of 1.5 × 10^5^ per well and allowed to grow overnight. To assess the cytotoxicity of the carbon nanotubes, 2D culture of B2B cells were exposed to various MWCNT treatments for 48 h. Non-floating cells were then collected and cell number was counted. MWCNT and S-MWCNT-C concentrations of 50 ng/mL and 5 μg/mL were selected for the entire study. 50 ng/mL was determined by converting the MWCNT dosage in mice (4 mg/kg) to *in vitro* concentration (50 ng/mL) based on the ratio of cell number in the animal lung to the cell number in 6-well-plate^26^,^27^,^28^. 5 μg/mL was determined based on IC50 measurement of cell viability *in vitro*.

### Brunauer–Emmett–Teller (BET) Measurement and X-ray Diffraction Measurement

The Brunauer–Emmett–Teller (BET) specific surface areas analysis was performed with Micromeritics TriStarII 3020. All samples were degassed in ultrahigh purity nitrogen for 10 min at room temperature, then 20 min at 90 degree Celcius and then for 150 min at 200 degree Celcius. The specific surface areas were determined by an 11-point BET measurement with liquid nitrogen as adsorptive. The relative pressures (P/P0) range is from 0.05–0.25. The X-ray Diffraction measurement was performed with Rigaku Ultima IV, using the Kα emission of a Cu X-ray source (λ = 1.5418 Å) at room temperature. In order to get an improved signal/background ratio, a thick aluminum foil was used instead of glass slide. The 2 θ ranged from 10° to 90°, where θ is the diffraction angle.

### Size and Surface Charge

Size and surface charges of carbon nanotubes were measured by dynamic light scattering (DLS) and zeta potential measurement (NanoBrook, Brookhaven Instruments Corp) following manufacture recommended procedure. MWCNT were suspended in dispersion medium and fully sonicated until there were no visible aggregates. For effective diameter, samples were diluted in cell culture medium and measured at a fixed scattering angle of 90° at 25 °C. For zeta potential, samples were diluted in deionized water, and measured by Electrophoretic Light Scattering (ELS) at 25 °C.

### Transmission Electron Microscopy

Transmission electron microscopy was performed using a JEOL-2010 TEM. MWCNT were suspended in 200 prove ethanol at the concentration of 0.01 mg/mL. MWCNT were sonicated 20 min (10s-on, 10s-off, 20 min effective sonication). Then, a droplet of MWCNT solution was put on the copper wire mesh of TEM, dried in air and loaded for TEM imaging.

### Sedimentation Test

Sedimentation test was performed following the method described previously^62^. Briefly, MWCNT were prepared at a series of concentrations in PBS and centrifuged for different time periods. After centrifugation, the absorbance of supernatant was measured at 525 nm.

### Cell Culture

BEAS-2B (B2B) normal lung epithelial cells were obtained from the American Type Culture Collection (ATCC) (Manassas, VA). B2B cells were routinely cultured in RPMI 1640 media (Life Technologies, 11875119, Carlsbad, CA) supplemented with 10% fetal bovine serum (FBS, Life Technologies, 26140079, Carlsbad, CA) and Penicillin Streptomycin (Invitrogen, 1514108, Carlsbad, CA).

### Cellular Uptake

B2B cells were seeded into 12-well plates at the density of 0.75 × 10^5^ cells per well and incubated overnight. MWCNT and S-MWCNT-C were added to the cells at the concentration of 5 μg/mL after sonication. At 2, 5, 10, 24, 48 h post exposure, cells were lysed by NaOH (0.2 M) for 2–3 h and pipetted up and down until MWCNT were uniformly distributed in the lysate. The absorbance (525 nm) of the lysate was measured by microplate reader (Tecan Group Ltd), following a procedure described previously^63^. Each sample had three replicates and the mean ± SD was reported.

### Microtissue Seeding and Cell Proliferation

After 24 h exposure of MWCNT and S-MWCNT-C, B2B cells were detached by trypsin treatment and seeded into the microtissue array device. Briefly, the micropillar devices were sterilized in 70% ethanol for 15 min before cell seeding and then treated with 0.2% Pluronic F127 (BASF) to reduce the surface adhesiveness of the PDMS. Unpolymerized rat tail collagen type I (Corning) was neutralized by NaOH, mixed with treated or non-treated B2B cells and then seeded into the device at a constant cell number of 400,000 cells per device. Microtissue culture was maintained for 3 days under the same condition as 2D culture, and cell culture media was changed every 48 hours. The microtissue formation was monitored daily by counting the number of formed microtissues. Cell proliferation in the microtissue was measured by counting the cell number at day 1, 2 and 3. Hoechst solution (1:500 dilution in PBS) was used to label the nuclei of live cells in the microtissue for counting cell numbers.

### Cell Viability Measurement after Cabon Nanotube Treatment

Cell viability was measured both in 2D culture and in microtissue culture. In 2D culture viability study, B2B cells were treated with MWCNT and S-MWCNT-C for 48 h, cells were then detached by trypsin treatment. Viable B2B cells were counted using trypan blue exclusion method. For viability study in microtissue, B2B cells were first treated with MWCNT and S-MWCNT-C in 2D culture. At 24 h post exposure, cells were trypsinized, seeded into microtissue device and allowed to grow for another 24 h. Then the cell viability in microtissues was measured by live/dead kit (Life Technologies; L3224, Carlsbad, CA). Each sample had three replicates and the mean ± SD was reported.

### ROS Measurement after Cabon Nanotube Treatment

ROS measurement was performed both in 2D culture and in microtissue culture. For 2D ROS measurement, B2B cells were treated with MWCNT and S-MWCNT-C for 48 h, and then were washed with PBS twice. Fresh cell culture medium containing ROS reagent (5 μM, CellROX^®^ Reagent, Life Technologies) was added to cells. Cells were then incubated at 37 °C for 30 min. After treatment with ROS reagent, cells were collected and fixed with 4% paraformaldehyde. The fluorescence intensity (ex.485/em.535) was measured by flow cytometery (BD LSRFortessa, BD Biosciences, San Jose, CA). Carbon nanotube treated cell samples without ROS reagent were used as the control. Each sample had three replicates and the mean ± SD was reported.

For ROS measurement in microtissues, B2B cells were first treated with carbon nanotubes for 24 h in 2D culture, and then collected and seeded into microtissue device. At 48 h post exposure, microtissues were washed with PBS twice. Fresh cell culture medium containing ROS reagent (5 μM, CellROX^®^ Reagent, Life Technologies) was added and incubated for 30 min at 37 °C. After incubation, the microtissues were washed off and collected by centrifugation (5000 rpm, 5 min). Microtissues were re-suspended in 150 μL PBS, and the fluorescence intensity (ex.485/em.535) was measured by the microplate reader (Tecan Group Ltd). Fluorescence intensity was normalized by microtissue number. Hydrogen peroxide (100 μM, treated for 2 h) was used as the positive control. Each sample had three replicates and the mean ± SD was reported.

### Microtissue Contraction Force Measurement

The dog-bone structure of the microtissue formed by day 1 for all groups except for 5 ug/mL MWCNT group. The images of both bottom and top position of the micropillars were taken for consistent microtissue samples for three continuous days. Micropillar deflection is determined by comparing the deflected position of the pillar top with its base 25. The contraction force was calculated according to cantilever bending theory as F = kδ, where δ is the averaged deflection δ = (δ 1 + δ 2)/2 of the two micropillars and k = 0.9 μN/μm is the spring constant of the micropillar (Fig. 1C). The distance between the two micropillars is 500 μm. The Young’s modulus of the PDMS is 1.6 MPa. To monitor the microtissue formation and measure the contraction force, Olympus 1 × 81 motorized microscope with 10X objective was used to image individual microtissue. Each sample had 5 replicates and the mean ± SD was reported.

### Expression of miR-21 by qRT-PCR

After 3 days culture in microtissue array device, the microtissues were collected for RNA isolation using mirVana^TM^ miRNA Isolation Kit (Life Technologies, AM1560, Carlsbad, CA). To determine the expression of miR-21 in microtissues, the total RNA was first reverse transcribed into cDNA using the TaqMan MicroRNA reverse transcription kit (Life Technologies, 4366596). The qRT-PCR amplification of cDNA was then performed using TaqMan MicroRNA assay (Life Technologies; assay ID 000397). The miR-21 expression was determined by the ∆∆Ct method and normalized to RNU48 (Life Technologies; assay ID 001006), which was the endogenous control in the corresponding samples, and relative to the untreated control group. Each sample had three replicates and the mean ± SD was reported.

### Immunofluorescence and Microscopy

Microtissues were fixed with 1% paraformaldehyde in PBS, permeabilized with Triton X-100, incubated with primary antibodies against ZO-1/TJP1 tight junctions (40–2300, Thermo Fisher), E-cadherin (Abcam) or tubulin (Abcam), labeled with fluorophore-conjugated, anti-IgG antibodies (AlexaFluor, Invitrogen) and counterstained with Hoechst 33342 (Invitrogen). F-actin was detected using Alexa Fluor 488 Phalloidin. Confocal images of the microtissue were taken either on a ZEISS 710 laser scanning microscope or an Andor Technology DSD2 confocal unit coupled to an Olympus IX-81 motorized inverted microscope. Plan-Apochromat 10X or 20X air objectives were used and image stack was taken in 2 μm optical slices for all channels. The stack of images was then processed using the 3D Viewer tool in ImageJ (NIH) to obtain the projected 2D views.

## Additional Information

**How to cite this article**: Chen, Z. *et al*. Lung Microtissue Array to Screen the Fibrogenic Potential of Carbon Nanotubes. *Sci. Rep.* **6**, 31304; doi: 10.1038/srep31304 (2016).

## Supplementary Material

Supplementary Information
