# Supplementary material for: Lung Microtissue Array to Screen the Fibrogenic Potential of Carbon Nanotubes
Source: Sci Rep. 2016 Aug 11;6:31304. doi: 10.1038/srep31304 (PMC4980669; doi:10.1038/srep31304)
Supplement: Supplementary Information [file srep31304-s1.pdf]

Supplemental material

LUNG MICROTISSUE ARRAY TO SCREEN THE FIBROGENIC POTENTIAL OF CARBON NANOTUBES

Zhaowei Chen, Qixin Wang, Mohammadnabi Asmani, Yan Li, Chang Liu, Changning Li, Julian M. Lippmann, Yun Wu, Ruogang Zhao

Department of Biomedical Engineering, State University of New York at Buffalo

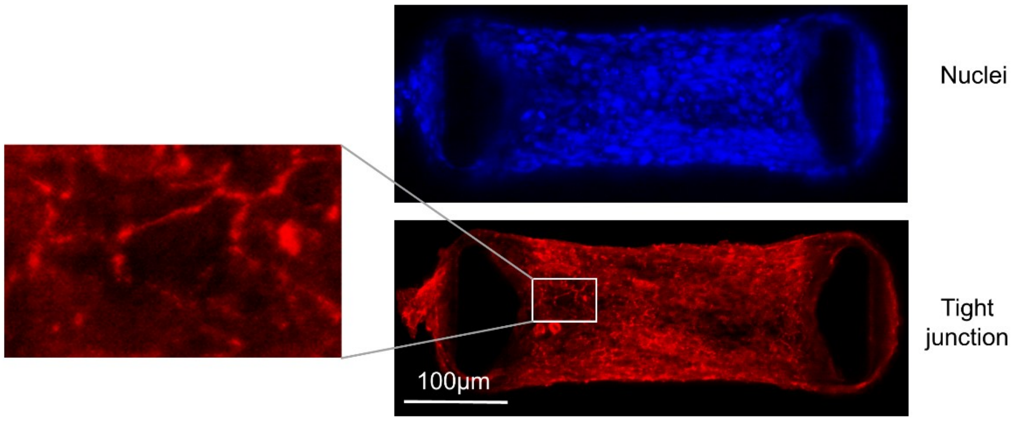

Figure S1. ZO-1 / TJP1 tight junction staining of a B2B cell populated microtissue at day 3. Tight junction in red and nuclei in blue.

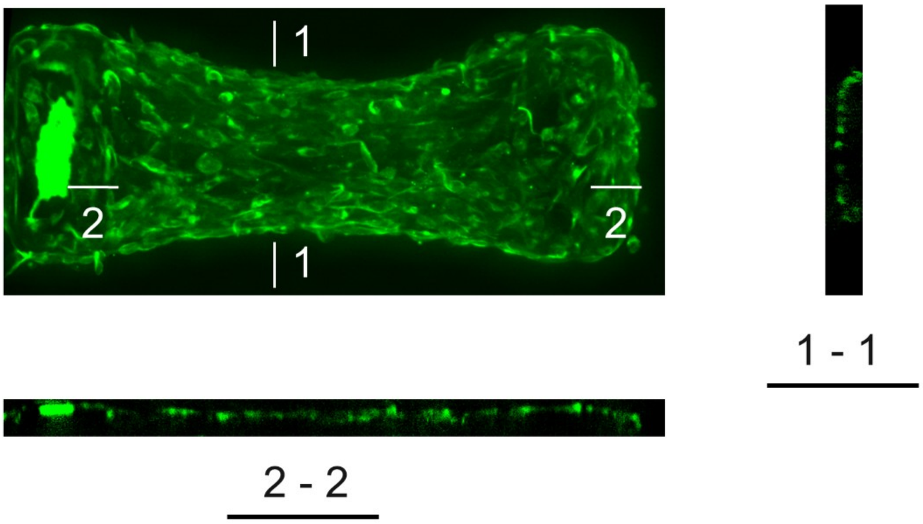

Figure S2. E-cadherin staining of a B2B cell populated microtissue at day 3. Cross-sectional views 1-1 and 2-2 show cells forming nearly a monolayer in the microtissue.

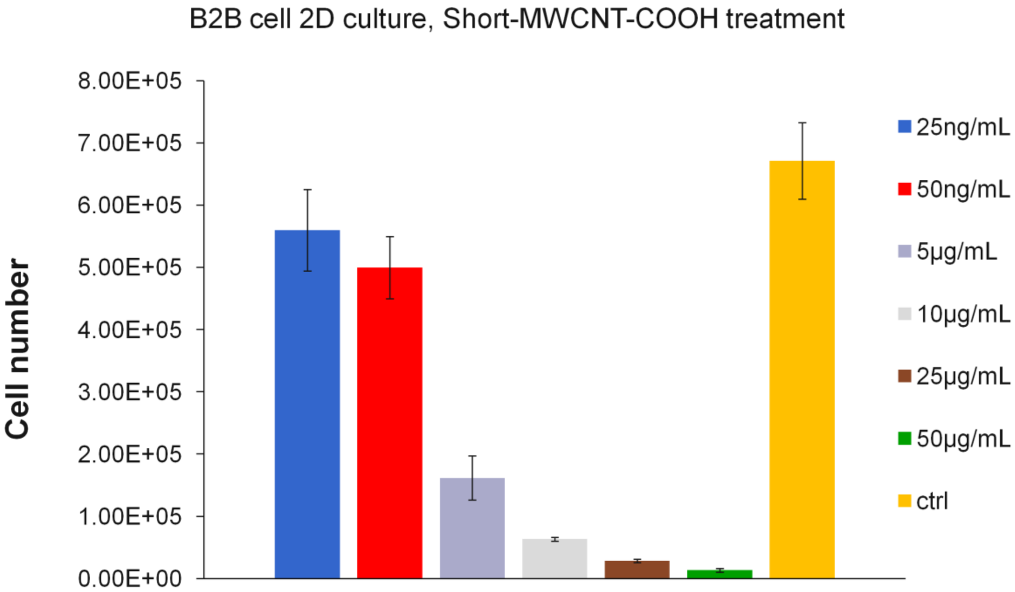

Figure S3. Cytotoxicity of B2B cells for a series of S-MWCNT-C concentrations. Increased S-MWCNT-C concentration induced increased level of cell loss as a result of cytotoxicity.

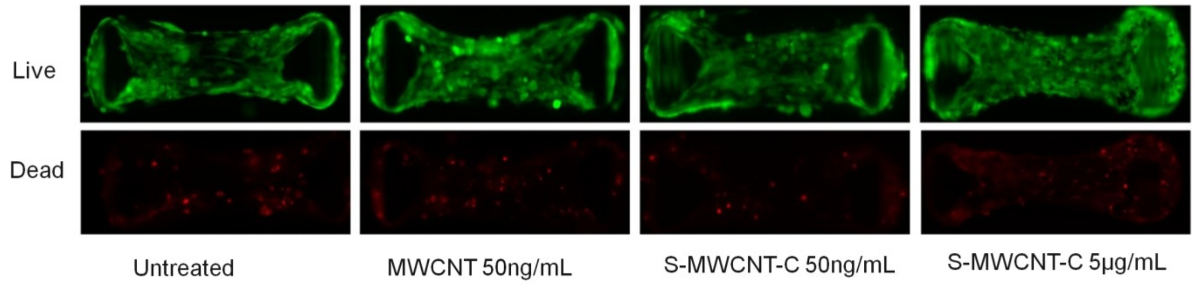

Figure S4. Live/dead staining of B2B cells in 3D microtissue after various carbon nanotube treatments. Only 2D cells that survived the carbon nanotube treatments and remained attached were trypsinzed and introduced into the 3D microtissues. Most of the cells were alive 1 day after seeding in the microtissue.

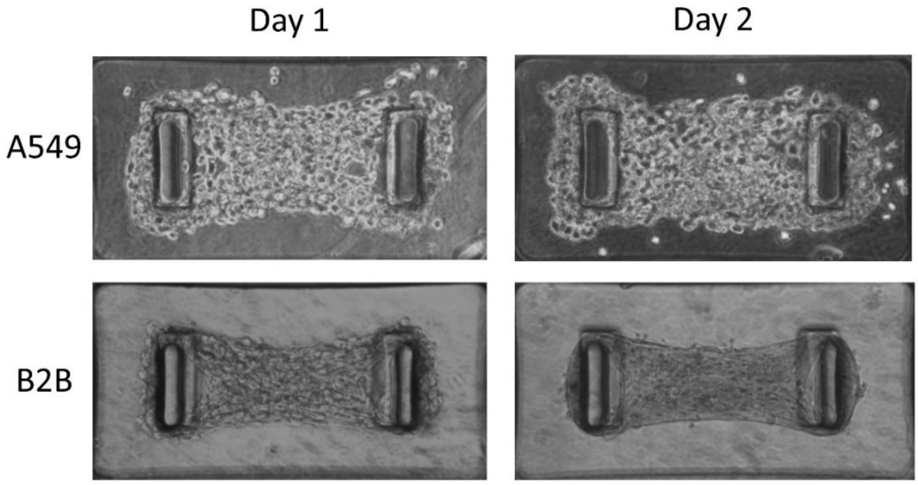

Figure S5. Microtissue structures for BEAS-2B cell (healthy) and A549 cell (cancerous) at day 1 and day 2. A549 cell populated microtissue represented a loose and non-stable morphology.

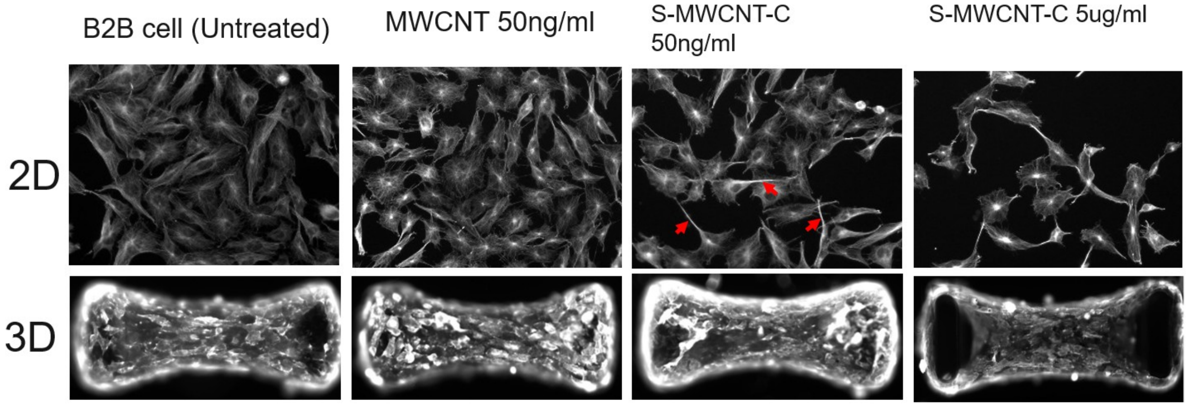

Figure S6. Microtubule morphology of B2B cells and microtissues under different carbon nanotube treatments. S-MWCNT-C treatments caused significant morphological change in B2B cells cultured in 2D. Cells formed long filopodia-like microtubule protrusions similar to the morphology of migrating cells (indicated by arrows). B2B cells and microtissues were stained with tubulin antibody (Abcam).

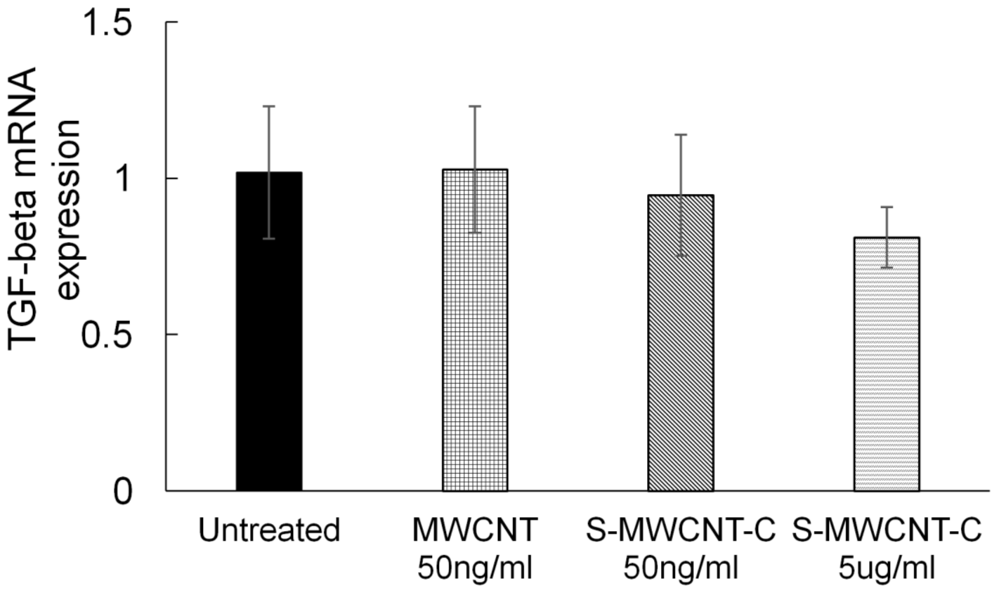

Figure S7. TGF-beta mRNA expression of microtissues under different carbon nanotube treatments. No significant difference was observed among treated groups and untreated control.
